# Interplay of cytokines in the pathophysiology of atopic dermatitis: insights from Murin models and human

**DOI:** 10.3389/fmed.2024.1342176

**Published:** 2024-03-25

**Authors:** Yuto Yamamura, Chisa Nakashima, Atsushi Otsuka

**Affiliations:** Department of Dermatology, Faculty of Medicine, Kindai University Hospital, Osaka, Japan

**Keywords:** AD, cytokines, clinical trials, Th2, Th22

## Abstract

The pathogenesis of atopic dermatitis (AD) is understood to be crucially influenced by three main factors: dysregulation of the immune response, barrier dysfunction, and pruritus. In the lesional skin of AD, various innate immune cells, including Th2 cells, type 2 innate lymphoid cells (ILC2s), and basophils, produce Th2 cytokines [interleukin (IL)-4, IL-5, IL-13, IL-31]. Alarmins such as TSLP, IL-25, and IL-33 are also produced by epidermal keratinocytes, amplifying type 2 inflammation. In the chronic phase, not only Th2 cells but also Th22 and Th17 cells increase in number, leading to suppression of filaggrin expression by IL-4, IL-13, and IL-22, which further deteriorates the epidermal barrier function. Dupilumab, which targets IL-4 and IL-13, has shown efficacy in treating moderate to severe AD. Nemolizumab, targeting IL-31RA, effectively reduces pruritus in AD patients. In addition, clinical trials with fezakinumab, targeting IL-22, have demonstrated promising results, particularly in severe AD cases. Conversely, in murine models of AD, several cytokines, initially regarded as promising therapeutic targets, have not demonstrated sufficient efficacy in clinical trials. IL-33 has been identified as a potent activator of immune cells, exacerbating AD in murine models and correlating with disease severity in human patients. However, treatments targeting IL-33 have not shown sufficient efficacy in clinical trials. Similarly, thymic stromal lymphopoietin (TSLP), integral to type 2 immune responses, induces dermatitis in animal models and is elevated in human AD, yet clinical treatments like tezepelumab exhibit limited efficacy. Therapies targeting IL-1α, IL-5, and IL-17 also failed to achieve sufficient efficacy in clinical trials. It has become clear that for treating AD, IL-4, IL-13, and IL-31 are relevant therapeutic targets during the acute phase, while IL-22 emerges as a target in more severe cases. This delineation underscores the necessity of considering distinct pathophysiological aspects and therapeutic targets in AD between mouse models and humans. Consequently, this review delineates the distinct roles of cytokines in the pathogenesis of AD, juxtaposing their significance in human AD from clinical trials against insights gleaned from AD mouse models. This approach will improve our understanding of interspecies variation and facilitate a deeper insight into the pathogenesis of AD in humans.

## 1 Introduction

Atopic dermatitis (AD) is a chronic, relapsing inflammatory dermatosis characterized by pruritic, erythematous, and edematous lesions. Predominantly manifesting in early childhood, the condition exhibits a variable incidence across different ages. The clinical presentation of AD is marked by episodic exacerbations and remissions, with affected individuals frequently presenting with xerosis, which exacerbates the itch-scratch cycle (1). The pathogenesis of AD is currently understood to be crucially influenced by three main factors: dysregulation of the immune response, barrier dysfunction, and pruritus (2). The underlying immune dysregulation in AD is characterized by an overactive T helper cell type (Th) 2 response. Additionally, mutations in the filaggrin gene, which plays a vital role in skin barrier function, are a primary cause of barrier dysfunction (3). Approximately 10–30% of AD patients exhibit mutations in the filaggrin gene (4). A meta-analysis of a genome-wide association study (GWAS) and GWAS revealed 31 loci associated with AD, including four loci with secondary independent signals (5). Several AD risk loci supported existing findings, including the role of skin barrier and type 2 inflammation in AD pathogenesis. In addition, it notes the identification of rare protein-coding variations contributing to AD heritability, including in genes such as interleukin (IL) 4 receptor (R), IL13, Janus kinase (JAK)1, JAK2, and TYK2, plus novel candidate genes.

In the lesional skin of AD, various innate immune cells, including Th2 cells, type 2 innate lymphoid cells (ILC2s), and basophils, produce Th2 cytokines (IL-4, IL-5, IL-13, IL-31). Alarmins such as thymic stromal lymphopoietin (TSLP), IL-25, and IL-33 are also produced by epidermal keratinocytes, amplifying type 2 inflammation (6). These cytokines further decrease the expression of barrier-associated proteins such as filaggrin, loricrin, and involucrin, leading to impaired barrier function (7).

Pruritus is one of the most prominent features of AD, although the mechanisms underlying AD-associated pruritus are not as well understood as those for barrier dysfunction and immune activation. Recently, several cytokines, including TSLP, IL-4/13, and IL-31, have been reported to be involved in AD-associated pruritus, and biological agents targeting these cytokines have been shown to improve pruritus in AD patients (8).

**FIGURE 1:**
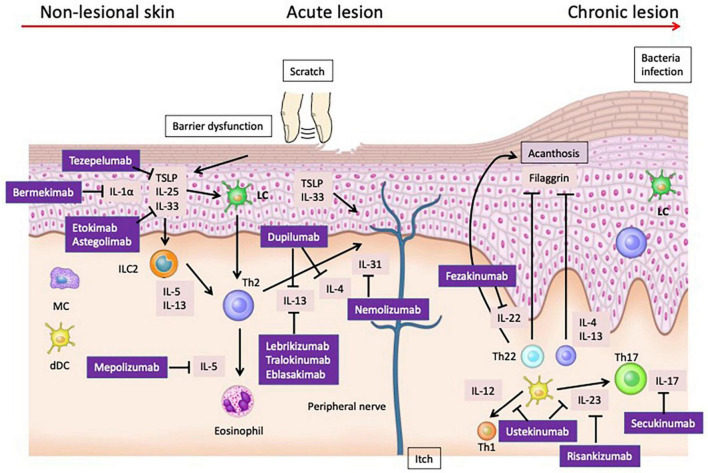
Pathogenesis and Therapeutic Targets of Atopic Dermatitis: Insights from Mouse Models and Human Specimens. The barrier dysfunction that occurs in the early stages of AD facilitates the penetration of allergens into the skin, and damaged keratinocytes produce cytokines such as TSLP, IL-25, and IL-33. These cytokines activate Th2 cells and ILC2s, leading to the production of Th2 cytokines. Additionally, TSLP matures Langerhans cells (LCs) in the epidermis and induces Th2 cells. During the acute phase, the skin barrier continues to deteriorate further. Damaged keratinocytes release various chemokines such as CCL17/thymus and activation-regulated chemokine and CCL22/macrophage-derived chemokine, as well as cytokines like TSLP, IL-1β, IL-25, and IL-33. These mediators activate ILC2 and Th2 cells at the lesion site. ILC2 cells produce IL-5 and IL-13, while Th2 cells produce IL-4, IL-13, IL-31, and IL-5. In the chronic phase, not only Th2 cells but also Th22 and Th17 cells increase in number, leading to suppression of filaggrin expression by IL-4, IL-13, and IL-22, further deteriorating the epidermal barrier function. Additionally, IL-4 and IL-13 suppress the production of AMPs, weakening the barrier function against microbes. Th17 and Th22 cells also produce IL-22, which induces epidermal thickening.

While numerous mouse models have been utilized to study AD and have significantly contributed to our understanding of the disease, it is important to note that the roles of various cytokines may not be completely identical between mice and humans. We searched PubMed and ClinicalTrials.gov from database inception to September 2023. Searches were adapted for each database, using keywords that included a combination of terms related to atopic dermatitis and clinical trial. We targeted biological agents undergoing phase 2 trials for AD. We searched for papers involving cytokines targeted by biological agents and relevant to the pathophysiology of AD, dividing the papers into mouse and human data (Table 1). In this review, we discuss the role of cytokines in the pathophysiology of separately for humans and mice and summarize their effects in clinical trials (Figure 1).

**TABLE 1:** Clinical trials and their outcomes in atopic dermatitis treatment.

Targeted cytokines	Agent	Mechanism	Description	Number of patient (Age)	Endpoint	Intervention arm	Results	ClinicalTrials.gov identifier	Development status	References
IL-33	Etokimab	Anti-IL-33	Monoclonal	302 (18 to 75 Years)	Week 16	Etokimab 600 mg/300 mg SC Q4W	Percent change in EASI Score: −44.56 (7.811)/−47.40 (6.091)/−55.70 (6.206)/−41.63 (6.707)/−49.38 (7.124)	NCT03533751	Phase 2 completed	(25)
						Etokimab 300 mg/150 mg SC Q4W				
						Etokimab 300 mg/150 mg SC Q8W				
						Etokimab 20 mg SC Q4W				
						Placebo				
IL-33	Astegolimab	Anti-IL-33		65 (18 to 75 Years)	Week 16	A loading dose of 245 mg SC MSTT1041A, followed by 490 mg of SC MSTT1041A every 4 weeks (Q4W)	Percent change of total EASI score: −58.24/−51.47	NCT03747575	Phase 2 completed	(26)
						Placebo				
TSLP	Tezepelumab	Anti-TSLP	Human monoclonal	251 (18 to 75 Years)	Week 16	Tezepelumab 420 mg Q2W	Number of participants with IGA score of 0 or 1: 5(7.2%)/2(3.2%)/4(6.5%)/2(3.2%)	NCT03809663	Phase 2 completed	(44)
						Tezepelumab 280 mg Q2W				
						Tezepelumab 210 mg Q4W				
						Placebo				
IL-1α	Bermekimab	Anti-IL-1α	Humanized monoclonal	6 (18 to 65 Years)	Week 16	Bermekimab 1200 mg IV	Percentage of participants with EASI-75: 0/0/0	NCT04990440	Phase 2 completed	
						Bermekimab 800 mg IV				
						Placebo				
IL-5	Mepolizumab	Anti- IL-5	Humanized monoclonal	34 (18 to 70 ears)	Week 16	Mepolizumab 100 mg SC	Number of participants with IGA score of 0 or 1 and at Least a 2- Grade Improvement: 2(11.1%)/0(0%)	NCT03055195	Phase 2 completed	(64)
						Placebo				
IL-4	Dupilumab	Anti-IL-4 receptor α	Human monoclonal	838 (18 Years and older)	Week 12	Dupilumab 300 mg + Oral placebo	IGA 0 or 1 and reduction (> = ) 2 points rate: 36.5/48.4/36.6/14.0	NCT03720470	Phase 3 completed	(86)
						PF-04965842 200 mg + Placebo injection	EASI response > = 75 percent (%) improvement rate: 58.1/70.3/58.7/27.1			
						PF-04965842 100 mg + Placebo injection				
						Placebo				
				740 (18 Years and older)	Week 16	Dupilumab qw + Topical corticosteroids	IGA 0 or 1 and reduction (> = ) 2 points rate: 39/39/12	NCT02260986	Phase 3 completed	(87)
						Dupilumab q2w + Topical corticosteroids	EASI response > = 75 percent (%) improvement rate: 64/69/23			
						Placebo + Topical corticosteroids				
				251 (12 to 17 Years)	Week 16	Dupilumab 200 or 300 mg Q2W	IGA 0 or 1 and reduction (> = ) 2 points rate: 24.4/17.9/2.4	NCT03054428	Phase 3 completed	(88)
						Dupilumab 300 mg Q4W	EASI response > = 75 percent (%) improvement rate: 41.5/38.1/8.2			
						Placebo				
				294 (12 to 17 Years)	Week 52		Proportion of patients achieving IGA 0/1 (%): 42.7	NCT02612454	Phase 3 completed	(89)
							Proportion of patients achieving EASI 75 (%): 81.2			
							Mean % change in EASI from PSBL: −83.5			
							Mean % change in SCORAD from PSBL: −65.0			
				367 (6 to 11 Years)	Week 16	Dupilumab q2w + Topical corticosteroids	IGA 0 or 1 and reduction (> = ) 2 points rate: :32/33/11	NCT03345914	Phase 3 completed	(90)
						Dupilumab q4w + Topical corticosteroids	EASI response > = 75 percent (%) improvement rate: 67.2/69.7/26.8			
						Placebo + Topical corticosteroids				
				202 (6 Months to 5 Years)	Week 16	Dupilumab q4w + Topical corticosteroids	IGA 0 or 1 and reduction (> = ) 2 points rate: 28/4	NCT03346434	Phase 3 completed	(91)
						Placebo + Topical corticosteroids	EASI response > = 75 percent (%) Improvement rate: 53/11			
IL-13	Lebrikizumab	Anti-IL-13	Humanized monoclonal	212 (18 to 75 Years)	Week 12	Lebrikizumab + TCS	IGA 0 or 1 and reduction (> = ) 2 points rate: :33/19	NCT02340234	Phase 2 completed	(93)
						Placebo + TCS	EASI response > = 50 percent (%) improvement rate: 82/52			
				280 (18 Years and older)	Week 16	Lebrikizumab 250 mg q2w	IGA 0 or 1 and reduction (> = ) 2 points rate: :44.6/33.7/26.6/15.3	NCT03443024	Phase 2 completed	(96)
						Lebrikizumab 250 mg q4w	EASI response > = 75 percent (%) improvement rate: 60.6/56.1/43.3/24.3			
						Lebrikizumab 125 mg q4w				
						Placebo q2w				
				206 (12 to 17 Years)	Week 52	Lebrikizumab 500 mg loading doses at baseline and Week 2, followed by 250 mg Q2W	IGA 0 or 1 and reduction (> = ) 2 points rate: 62.6	NCT04250350	Phase 3 completed	(97)
							EASI response > = 75 percent (%) improvement rate: 81.9			
							EASI response > = 50 percent (%) improvement rate: 94.4			
IL-13	Tralokinumab	Anti-IL-13	Human monoclonal	130 (12 Years and older)	Week 16	Tralokinumab + TCS	IGA 0 or 1 and reduction (> = ) 2 points rate: 27/12	NCT05194540	Phase 3 completed	(93)
						Placebo + TCS	EASI response > = 50 percent (%) improvement rate: 73/52			
				794 (18 Years and older)	Week 16	Tralokinumab 300 mg q2w	IGA 0 or 1 and reduction (> = ) 2 points rate: 15-8/7-1 (ECZTRA1), 22-2/10-9 (ECZTRA2)	NCT03131648 (ECZTRA1), NCT03160885 (ECZTRA2)	Phase 3 completed	(94)
						Placebo q2w	EASI response > = 75 percent (%) improvement rate: 25-0/12-7 (ECZTRA1), 33-2/11–4 (ECZTRA2)			
				301 (12 to 17 Years)	Week 16	Tralokinumab 300 mg q2w	IGA 0 or 1 and reduction (> = ) 2 points rate: 17. 5/21.4/4.3	NCT03526861	Phase 3 completed	(95)
						Tralokinumab 150 mg q2w	EASI response > = 75 percent (%) improvement rate: 27.8/8.6/6.4			
						Placebo q2w				
IL-31	Nemolizumab	Anti- IL-31 receptor	Humanized monoclonal	215 (12 Years and older)	Week 16	Nemolizumab 60 mg q4w	Change in VAS score for pruritus to Week 16: −42.8/−21.4	NCT03985943	Phase 3 completed	(117)
						Placebo q4w	Change in VAS score for pruritus to Day 15: −30.4/−11.1			
							Change in EASI score : −45.9/−33.2			
IL-22	Fezakinumab	Anti- IL-22	Monoclonal	60 (18 to 75 Years)	Week 12	ILV-094	Percentage change in SCORAD: −18.8/−11.7	NCT01941537	Phase 2 completed	(135)
						Placebo				
IL-17	Secukinumab, MOR106	Anti-IL-17A, Anti-IL-17C	Monoclonal	41 (18 Years and older)	Week 16	Secukinumab (300 mg) via	Fold-change in Epidermal thickness of Lesional skin: 1.18/1.15 (for Extrinsic AD)	NCT02594098	Phase 2 completed	(1, 51, 152)
						Placebo	Fold-change in Epidermal Thickness of Lesional skin: −1/1.5 (for Intrinsic AD)			
IL-23p19	Risankizumab	Anti-IL-23A	Humanized monoclonal	172 (12 Years and older)	Week 16	Risankizumab 300 mg	Percentage of participants achieving at least EASI-75: 21.7/24.6/11.8	NCT03706040	Phase 2 completed	(165)
						Risankizumab 150 mg				
						Placebo				
IL-12/23	Ustekinumab	Anti-IL-12 and -IL-23	Human monoclonal	79 (20 to 65 Years)	Week 12	Ustekinumab 90 mg	Percent Change in EASI score: −39.39/−38.62/−37.54	NCT01945086	Phase 2 completed	
						Ustekinumab 45 mg				
						Placebo				

## 2 Non-lesional skin

The barrier dysfunction that occurs in the early stages of AD facilitates the penetration of allergens into the skin, and damaged keratinocytes produce cytokines such as TSLP, IL-25, and IL-33. These cytokines activate Th2 cells and ILC2s, leading to the production of Th2 cytokines (9). Additionally, TSLP matures Langerhans cells (LCs) in the epidermis and induces Th2 cells (10).

Thus, in AD, subclinical inflammation is present even in the non-lesional skin at the initial stages of the disease, characterized by an increased expression of Th2 cytokines (IL-4, IL-13, IL-31, TSLP, IL-5) mediated by Th2 cells and other immune cells.

A decrease in the diversity of the microbial community on the epidermis and a relative increase in *Staphylococcus aureus* (*S. aureus*) have also been observed, which further amplify the Th2 immune response (11). This complex interplay of factors contributes to the exacerbation of AD symptoms and the perpetuation of the inflammatory state (12).

### 2.1 IL-33

IL-33, a member of the IL-1 family, is a multifaceted cytokine that affects various cell types including Th2 cells, mast cells, basophils, eosinophils, macrophages, dendritic cells (DCs), and ILC2s. It is primarily produced by epithelial cells, fibroblasts, and endothelial cells. IL-33 binds to a heterodimeric receptor consisting of ST2 (also known as IL-1RL1) and the IL-1 Receptor Accessory Protein (IL-1RAcP), leading to the activation of the NF-kB and mitogen-activated protein kinases (MAPK) (Extracellular signal-Regulated Kinase (ERK), p38, c-Jun N-terminal kinase (JNK)) signaling pathways (13). Full-length IL-33, released from epithelial cells, is cleaved into its active form by various proteases and allergens (14). The IL-33 receptor, ST2, is expressed on immune cells such as Th2 cells, mast cells, eosinophils, and basophils. Consequently, IL-33 acts as a sensitive sensor to protease allergens, promoting the proliferation, activation, and recruitment of Th2 cells (15).

#### 2.1.1 Roles in mouse AD model

In a mouse AD model, IL-33 overexpression in the skin of IL-33 Transgenic (Tg) mice spontaneously activated ILC2s and induced a pruritic dermatitis like AD, suggesting that IL-33 is involved in the onset of AD (16). The inflammation induced by IL-33 in this model depends on a natural immune response mediated by ILC2s in coordination with basophils (17). Treatment with αIL-33 Antibody (Ab) in a Dinitrochlorobenzene (DNCB)-induced AD mouse model improved AD-like symptoms, reduced eosinophil and mast cell infiltration, and decreased serum Immunoglobulin E (IgE) levels (18). Additionally, propionate, a metabolic product of sebum, suppressed skin inflammation in a calcipotriol (MC903), a calcium analog of vitamin D3, -induced AD-like dermatitis mouse model by inhibiting IL-33 production in keratinocytes (19).

#### 2.1.2 Roles in human AD

In human AD, serum IL-33 levels correlate with clinical severity (20), and expressions of IL-33 and its receptor components, ST2 and IL-1RAcP, are increased in lesional skin. Skin lesions from AD patients, after application of house dust mite (HDM) or staphylococcal enterotoxin B (SEB), also showed increased expressions of IL-33 and ST2 (21). Pathogenic factors from *S. aureus* directly promote IL-33 production from human keratinocytes and destroy skin barrier functions, suggesting that these factors from S. aureus adhering to the skin may initiate type 2 inflammation via IL-33 in AD (22). *In vitro* stimulation of human basophils with IL-33 induces the production of IL-4 and IL-13 (23). IL-33 also activates human eosinophils, promoting their adhesion and survival (24, 25).

Despite these findings suggesting the involvement of IL-33 in the pathophysiology of human AD, clinical trials have yet to prove the efficacy of anti-IL-33 antibodies. In a phase IIa trial, a single systemic dose of etokimab, an IgG1 monoclonal antibody against IL-33, provided rapid and sustained clinical benefits in 12 adult patients with moderate to severe AD (26). However, a subsequent phase II placebo-controlled trial with 302 participants did not show the efficacy of etokimab compared to the placebo at 16 weeks, based on the Eczema Area and Severity Index (EASI) change rate. Additionally, a fully human IgG2 monoclonal antibody, astegolimab, did not show significant differences compared to placebo in a phase II trial (27). A randomized placebo-controlled phase II trial of astegolimab in adults with moderate to severe AD also failed to show efficacy (28). These results suggest that IL-33 may play a limited role in human AD compared to mouse models.

### 2.2 TSLP

Thymic stromal lymphopoietin binds to a heterodimeric receptor composed of the TSLP receptor (TSLPR) chain closely related to the IL-7 receptor (IL-7R) α chain and the common receptor γ chain (γc), exhibiting biological activity across a wide range of cell types. While TSLPR alone has a low affinity for TSLP, the affinity significantly increases when TSLPR and IL-7Rα bind together, forming a high-affinity binding site for TSLP and inducing signal transduction (29). TSLP is extensively studied as a master regulator of type 2 immune responses occurring at barrier surfaces such as skin, lungs, and intestines. TSLP produced by epithelial cells activates DCs expressing the TSLPR, leading to the induction of functional Th2 cells (30). Furthermore, in both acquired and innate immunity, basophils and ILC2s play crucial roles downstream of TSLP (31).

#### 2.2.1 Roles in mouse AD model

In mouse models of AD, topical application of MC903 induces TSLP expression in epidermal keratinocytes, triggering AD-like dermatitis (32). Overexpression of TSLP in skin-specific manners results in a phenotype resembling AD, including infiltration of inflammatory cells in the dermis, development of eczematous lesions, a dramatic increase in Th2 CD4+ T cells expressing skin-homing receptors, and elevated serum IgE levels (33). LCs acting as antigen-presenting cells in the epidermal signaling pathway via TSLP-TSLPR play a crucial role in inducing Th2 immune responses in Ovalbumin (OVA)-induced mouse AD models (10). TSLP promotes peripheral basophil proliferation, and basophils expressing TSLPR restore Th2 immunity in mice (34). In the aforementioned MC903-induced mouse AD model, ILC2s play a significant role in inflammation onset (35). Additionally, IL-13 induces AD through a TSLP-dependent mechanism (36). Concerning pruritus in AD, injecting TSLP into the skin of mouse cheeks triggers scratching behavior dependent on IL-7Rα and primary afferent neurons. This response is due to the direct expression of TSLPR on primary afferent sensory neurons, requiring the Transient receptor potential cation channel subfamily A member 1 (TRPA1) ion channel for TSLPR activation (37). These data identify TSLP as a novel endogenous pruritogen, suggesting that keratinocyte-derived TSLP could be a therapeutic target for pruritus in AD. Overall, an increase in TSLP levels is known to be involved in the enhancement of Th2 immune responses (38).

#### 2.2.2 Roles in human AD

In humans with AD, TSLP serum levels are significantly higher in both children and adults compared to healthy individuals (39, 40). TSLP is expressed in keratinocytes at acute and chronic AD lesion sites but not in non-lesion skin of AD patients, lesion sites of patients with nickel-induced allergic contact dermatitis (ACD), or cutaneous lupus erythematosus (41). In AD patients, circulating CD4+ T cells express higher levels of TSLPR compared to healthy individuals, and the levels of circulating TSLPR+ CD4+ T cells correlate with serum Thymus and activation-regulated chemokine/chemokine ligand 17 (TARC/CCL17) and IgE levels, as well as eosinophil counts (42). When inflammation in AD is exacerbated, *S. aureus* produces proteases and invades the dermis of AD patients, leading to increased production of type 2 cytokines such as TSLP, IL-4, and IL-13 (43). The cell wall components of *S. aureus* also signal through toll-like-receptor 2/6, inducing TSLP production in keratinocytes (44). These findings suggest that TSLP is involved in the pathogenesis of AD.

In clinical trials, tezepelumab, an anti-TSLP monoclonal antibody, demonstrated good safety and tolerability profiles, with linear pharmacokinetics in both healthy individuals and AD subjects (45). However, in a Phase II trial comparing tezepelumab and topical corticosteroids (TCS) combination therapy to placebo and TCS, although there were numerical improvements in the proportion of patients achieving EASI50 at week 12 and exploratory endpoints, and further improvement at week 16, no significant difference was observed (NCT03809663) (46). While tezepelumab has proven efficacy in asthma, its effects in AD were insufficient. These results suggest that targeting TSLP for the treatment of AD in humans may have limited potential.

### 2.3 IL-1α

The IL-1 family plays a crucial role in the proper functioning and control of the innate immune system, connecting innate and adaptive immune responses (47). This complex family consists of several cytokines, receptors, and co-receptors, all working together in balance to maintain homeostasis (47). Dysregulation of these processes can lead to tissue inflammation and contribute to the pathogenesis of common inflammatory skin diseases such as psoriasis, pustular sweat gland inflammation, and AD (47).

The IL-1 family of cytokines comprises 11 cytokine members, with 7 agonists (IL-1α, IL-1β, IL-18, IL-33, IL-36α, IL-36β, IL-36γ) and 4 antagonists [IL-1 receptor antagonist (Ra), IL-36Ra, IL-37, IL-38] (48). Based on their structural and functional characteristics, these cytokines are further classified into four subfamilies: IL-1, IL-18, IL-33, and IL-36. Both IL-1α and IL-1β are pro-inflammatory cytokines. IL-1α is constitutively or inducible expressed in hematopoietic immune cells and other cell types such as intestinal epithelial cells and skin keratinocytes (49). IL-1α expression can be induced by inflammatory stimuli, leading to binding to IL-1R1 and the subsequent expression of inflammatory genes targeting type 1 or 17 immune responses. This results in the recruitment and activation of T cells, DCs, neutrophils, and monocytes/macrophages, further releasing inflammatory cytokines and chemokines, forming a self-amplifying inflammatory loop (50). On the other hand, IL-1β is primarily circulating, and its expression is inducible only in monocytes, macrophages, and DCs (50). The antagonist IL-1Ra competes with IL-1α and IL-1β for binding to the IL-1R1 receptor, exerting an anti-inflammatory effect (51).

#### 2.3.1 Roles in mouse AD model

In a study exploring the anti-inflammatory effects of topical Tetracycline (TET) on AD in a mouse model, TET was found to suppress the expression of inflammatory cytokines, including IL-1β, in skin lesions. High levels of these cytokines were observed in the AD group, indicating a role for IL-1β in the inflammatory process of AD (52). Another study showed that skin and keratinocytes from mice with filaggrin deficiency had upregulated expression of IL-1β and IL-1RA mRNA (53).

#### 2.3.2 Roles in human AD

Bermekimab, an inhibitor of IL-1α, showed promising results in a Phase II open-label trial with hidradenitis suppurativa (HS) patients, demonstrating a significant reduction in inflammatory lesions even after anti-tumor necrosis factor (TNF) therapy failure without severe drug-related adverse events (51, 54, 55). Contrasting efficacy has been reported for Canakinumab, a human monoclonal antibody targeting IL-1β, in case reports of HS (56, 57). While effective in severe cases of pustular psoriasis (58), it was reported ineffective in two patients with severe palmoplantar pustulosis (59).

From the above, it can be inferred that the IL-1 family is involved in the pathogenesis of inflammatory skin diseases, including AD. However, two Phase II trials on Bermekimab, an anti-IL-1αAb, for moderate to severe adult AD patients (NCT04990440 and NCT04021862) were discontinued due to lack of efficacy, suggesting a limited role of IL-1α in human AD.

## 3 Acute lesion

During the acute phase, the skin barrier continues to deteriorate further. Damaged keratinocytes release various chemokines such as CCL17/thymus and activation-regulated chemokine and CCL22/macrophage-derived chemokine, as well as cytokines like TSLP, IL-1β, IL-25, and IL-33 (60). These mediators activate ILC2 and Th2 cells at the lesion site. ILC2 cells produce IL-5 and IL-13 (61), while Th2 cells produce IL-4, IL-13, IL-31, and IL-5.

At the site of the lesion, there is an infiltration of CD4+ cells and an increase in the number of DCs, including LCs. DCs extend their dendritic processes beyond tight junctions to capture antigens. Furthermore, IL-4 and IL-13 promote IgE class-switching in B cells. In addition to these processes, various chemokines produced by keratinocytes at the inflammation site are involved in recruiting immune cells to the lesion. For example, eosinophils are activated by IL-5.

Regarding pruritus, IL-4 and IL-13 are suggested to act on IL-4Ra expressed on peripheral nerves, transmitting chronic pruritus through the Janus kinase (JAK) 1 signaling pathway. IL-31 acts on IL-31R expressed on peripheral nerves, eliciting pruritus (60). IL-31 is considered a primary cause of pruritus in AD. TSLP induces the expression of CD134 (OX40) ligand on DCs, which binds to OX40 on T cells, further stimulating the production of IL-4, IL-13, IL-5, and the pruritus-specific cytokine, IL-31.

### 3.1 IL-5

IL-5 plays a critical role in the development, survival, and proliferation of eosinophils (62). The primary producers of IL-5 are Th2 cells and ILC2, but mast cells, eosinophils, basophils, epithelial cells, and smooth muscle cells also produce IL-5 (62). IL-5 binds to a heterodimeric receptor composed of IL-5R subunit α (IL-5Rα) and the common subunit β (βc) (62). The βc subunit is also associated with IL-3Rα and the granulocyte-macrophage colony-stimulating factor (GM-CSF) Rα. In concert with IL-3 and GM-CSF, IL-5 promotes the proliferation, differentiation, and activation of eosinophils (62).

#### 3.1.1 Roles in mouse AD model

In transgenic mice overexpressing IL-5, specifically in keratinocytes, there is an infiltration of eosinophils in the epidermis, displaying an AD-like phenotype (63). Additionally, these mice show a significant increase in the number of sensory neurons in the epidermis, suggesting a potential involvement of IL-5 in the branching of nerve cells in AD (63).

#### 3.1.2 Roles in human AD

In humans, stimulation of peripheral blood mononuclear cells from children with AD using house dust mite extract resulted in IL-5 production correlating with the severity of AD (64). Moreover, infusion of the anti-IL-5Ab mepolizumab in AD patients significantly reduced eosinophil infiltration at the allergen injection site in the skin after 6 and 48 h and significantly reduced the number of tenascin-immunoreactive cells, a marker for repair and remodeling, after 48 h (65). These results suggest that IL-5 plays a significant role in the development of AD. However, mepolizumab did not demonstrate sufficient efficacy in AD patients (NCT03055195) (66), while it showed significant efficacy in specific subtypes of asthma patients (67, 68), suggesting that the role of eosinophils in the pathogenesis may differ between AD and asthma.

### 3.2 IL-4/13

IL-4 and IL-13 are representative cytokines of type 2 inflammatory responses and share many common functions. IL-4 is involved in Th2 differentiation and controls lymphocyte functions such as IgE synthesis in B cells. On the other hand, IL-13 is an effector cytokine that controls the construction of smooth muscle cells and mucus production in the airway epithelium in allergic asthma (69). Th2 cells, mast cells, eosinophils, and basophils all produce both IL-4 and IL-13 (70). ILC2s can produce IL-4, but generally at lower levels compared to their robust production of IL-13 (71).

IL-4 binds to either type 1 IL-4 receptor (IL-4R) or type 2 IL-4R. Type 1 IL-4R consists of IL-4Rα subunit and the common γ subunit of cytokine receptors. Type 2 IL-4R, on the other hand, consists of IL-4Rα and IL-13Rα1 chain (70). Therefore, type 2 IL-4R also functions as IL-13R. Hematopoietic/immune cells mainly express type 1 IL-4R, while type 2 IL-4R/IL-13R is ubiquitously expressed in non-hematopoietic cells and tissue-resident cells. Myeloid cells can express either type 1 or type 2 IL-4R. Because of the different distribution of IL-4R/IL-13R, IL-4 mainly functions in hematopoietic/immune cells, whereas IL-13 functions in non-hematopoietic cells and tissue-resident cells.

In the pathogenesis of AD, IL-4 and IL-13 are involved in (i) chemokine production, (ii) barrier function, (iii) pruritus, (iv) antimicrobial peptide (AMP) production, and (v) fibrosis. In terms of chemokine production, IL-4/IL-13 can induce various chemokines such as TARC/CCL17, CCL5, eotaxin-1/CCL11, and eotaxin-3/CCL26 either alone or in combination with other cytokines such as TNF-α or Interferon (IFN)-γ. These chemokines are highly expressed in the lesional skin of AD (72), and they recruit inflammatory cells such as T cells, eosinophils, and basophils to the skin lesions. In terms of barrier function, either IL-4 or IL-13 reduces the expression of barrier-associated molecules such as filaggrin, loricrin, and involucrin, leading to disruption of tight junctions and impaired ceramide production in the skin (73). IL-13 may directly or indirectly increase collagen deposition and fibrotic tissue remodeling (74), which is clinically observed in lichenified lesions of chronic AD.

#### 3.2.1 Roles in mouse AD model

Various genetically modified mice have demonstrated the importance of IL-4 or IL-13 in the development of AD. For example, mice overexpressing IL-4 or IL-13 in keratinocytes exhibit xerosis and pruritic dermatitis, major characteristics of human AD, accompanied by a type 2 immune response (75–77). IL-4 plays a crucial role in the control of epidermal homeostasis and the natural barrier function (78). In IL-4 transgenic mice, hundreds of dysregulated factors have been identified before and after the onset of skin lesions, with a significant increase in the expression of factors such as C-X-C motif chemokine ligand 5 (CXCL5), IL-1β, IL-24, IL-6, oncostatin M (OSM), prostaglandin-endoperoxide synthase 2 (PTGS2), Formyl Peptide Receptor 1 (FPR1), and Regenerating Islet-Derived Protein 3 Gamma (REG3γ) (79). Moreover, IL-4 and/or IL-13 have been proven to directly induce scratching behavior in mice (80).

#### 3.2.2 Roles in human AD

IL-4 and IL-13 inhibit the production of AMPs, human β-defensin (HBD)-2, and HBD-3 (81). This aligns with findings that AD patients exhibit lower expression levels of AMPs, such as cathelicidin (LL-37) and HBD-2 (82). These findings partially explain why AD patients are more susceptible to skin infections (83). IL-13 induces the expression of matrix metalloproteinase (MMP)-9 in human keratinocytes, acting on collagen IV in the basement membrane to promote cell movement and tissue remodeling (84, 85). In contrast, IL-13 downregulates MMP-13 expression in human fibroblasts, potentially leading to reduced collagen degradation and fibrosis observed in the thickened dermis of chronic lichenified AD lesions (86). Additionally, both mouse and human primary sensory neurons express receptors for IL-4 and IL-13 (87). Neurons pre-treated with IL-4 and IL-13 respond to sub-threshold concentrations of histamine and IL-31 (87).

Dupilumab, developed based on these findings, is a fully human monoclonal antibody against IL-4Rα. It binds to the IL-4Rα subunit of both type I and type II receptors, inhibiting both IL-4 and IL-13 mediated signaling pathways. It has shown significant efficacy in moderate to severe AD patients (88). The effectiveness of dupilumab has been demonstrated in studies involving adults and adolescents with AD (89, 90). Long-term administration in adolescents maintained effectiveness and showed a tolerable safety profile, highlighting the importance of continuous treatment for sustained efficacy (91). The q2w dosing regimen was found to be optimal for this age group (91). Dupilumab administration in younger AD patients (6 months to 11 years old) was effective and well-tolerated, with a safety profile consistent with that in older children and adults (92, 93).

A meta-analysis of 22 studies involving 3303 AD patients reported significant improvements in EASI scores and a high tolerance for dupilumab treatment, confirming its effectiveness in treating AD (94). Adverse events, such as conjunctivitis, were observed, but the treatment was generally well-tolerated (94). A 52-week retrospective study examined patients with moderate-to-severe atopic AD treated with dupilumab at labeled dosage (95). Patients were split into Group A (patients with significant comorbidities) and Group B (patients without significant comorbidities). Disease severity was measured using EASI, Pruritus-Numerical Rating Scale (P-NRS), and Dermatology Life Quality Index (DLQI) at baseline and weeks 4, 16, 24, and 52. The study included 263 patients, with 25 in Group A and 238 in Group B. Significant reductions in EASI, DLQI, and P-NRS were observed in both groups at each follow-up visit (*p* < 0.0001), with no notable differences between the groups. Safety outcomes were similar between the two groups. Serious side effects were not collected, and the main side effect was injection site reactions for both groups (Group A: 3, 12.0%; Group B: 41, 17.22%), followed by conjunctivitis (Group A: 2, 8.0%; Group B: 21, 11.34%). Another retrospective study demonstrated dupilumab’s effectiveness in treating adults with moderate to AD and chronic rhinosinusitis with nasal polyps (96). Using various measures, including EASI and 22-item Sino-Nasal Outcome Test, they observed significant improvements in both conditions at weeks 16 and 24. The long-term effectiveness and safety of dupilumab have been evaluated in patients with AD who also have comorbidities such as malignancy, severe renal insufficiency requiring dialysis, hepatitis B or C, AIDS, Parkinson’s disease, multiple sclerosis, or undergoing organ transplant (95). Based on this, targeted therapy against IL-4 and IL-13 is considered to have very high safety.

Other biological agents targeting the IL-13 signaling pathway, such as lebrikizumab and tralokinumab, have also demonstrated significant efficacy in adult AD patients (97). Tralokinumab has shown long-term efficacy and tolerability in adults and good tolerability in adolescents (98), supporting its value as a therapeutic for moderate to severe young AD (99). Lebrikizumab demonstrated rapid, dose-dependent effectiveness across a broad range of clinical symptoms in adults with AD and maintained a favorable safety profile in adolescents (100), significantly improving AD symptoms and quality of life (101).

IL-13 is considered a major mediator involved in the inflammation, epidermal barrier dysfunction, and pruritus associated with AD. Selective IL-13 inhibitors such as tralokinumab, lebrikizumab, and eblasakimab have shown promising efficacy in the treatment of moderate to severe AD (102). While their safety profiles are generally favorable, there is a heightened risk of conjunctivitis, necessitating monitoring (102). These findings collectively affirm the pivotal role of IL-4/13 in human AD.

### 3.3 IL-31

IL-31 is a member of the IL-6 cytokine family, predominantly produced by activated CD4+ T cells, particularly activated Th2 cells, as well as mast cells, macrophages, and DCs (103–107). The expression of IL-31 mRNA has been reported in various human tissues, including testes, bone marrow, skeletal muscle, and kidneys (103). The receptor for IL-31 is a heterodimer composed of IL-31 receptor A (IL-31RA) and OSM receptor (OSMR) (103). IL-31RA mRNA expression is observed in various tissues and cells, including testes, bone marrow, skin, dorsal root ganglia, activated monocytes, macrophages, DCs, eosinophils, basophils, and keratinocytes, while OSMR mRNA is broadly expressed in many tissues (107–109).

#### 3.3.1 Roles in mouse AD model

In the mouse AD model, IL-31 is implicated in skin pruritus as evidenced in the Fluorescein Isothiocyanate (FITC) and Dinitrofluorobenzene (DNFB)-induced contact dermatitis model, though it does not appear to be involved in inducing local skin inflammation (110). Moreover, repeated administration of IL-31 also increased the expression of IL-31RA and OSMR β in dorsal root ganglia, suggesting an upregulation of IL-31RA expression in dorsal root ganglion (DRG) neuron cell bodies by IL-31 (111). This correlation is further supported by enhanced scratching behavior observed upon continuous subcutaneous injection of IL-31 in mice (111). Additionally, a single dose of IL-31 in mice induced strong pruritus upon skin and intrathecal injection, with concentrations significantly increased in the skin of mice with atopic-like dermatitis, leading to persistent scratching behavior (19). This implication of IL-31 in pruritus and the promotion of scratching behavior was further corroborated in Nishiki-nezumi Cinnamon/Nagoya (NC/Nga) mice with dermatological lesions, serving as a model for AD (112, 113). Furthermore, transgenic mice overexpressing IL-31 developed severe pruritus and skin lesions, suggesting a role for IL-31 in allergic dermatitis (103). In a contrasting observation, IL-31RA knockout mice showed increased OSM-induced cytokine levels during airway sensitization and challenge (114). Finally, the administration of anti-IL-31 antibodies was found to improve scratching behavior in NC/Nga mice (115).

#### 3.3.2 Roles in human AD

IL-31, through the phosphorylation of signal transduction and activator of transcription (STAT)-1 and STAT-5, induces pro-inflammatory effects in activated human macrophages (116). The activation of ERK1/2 by IL-31 contributes to the underlying mechanism of Th1 cytokine IL-12 suppression in macrophages (116). Although IL-31 activates STAT-3 phosphorylation and enhances C-C motif chemokine 2 (CCL2) secretion in human primary keratinocytes, this phenomenon is not observed in AD keratinocytes with low TLR-2 expression, suggesting a potential link between the functional change of IL-31 and skin inflammation (117). Additionally, IL-31 is activated when the IL-31Rα receptor chain in primary human CD1c+ and monocyte-derived DCs is upregulated by IFN-γ stimulation, leading to a dose-dependent release of inflammatory mediators such as TNF-α, IL-6, CXCL8, CCL2, CCL5, and CCL22, causing skin inflammation (109). IL-31 is also present in eccrine sweat and activates keratinocytes to produce the inflammatory cytokine CCL2 (118). Human dorsal root ganglion neurons, many of which co-express TRP, Subfamily V, Member 1 (TRPV1), also express IL-31RA (19). Blocking TRPV1 *in vivo* interrupts IL-31 signaling (19). An increase in skin IL-31 may be associated with pruritus in diabetes mellitus (DM), and ongoing clinical trials aim to evaluate the systemic treatment effects on IL-31 and pruritus in DM (119). Staphylococcal superantigens have been shown to rapidly induce IL-31 expression in atopic patients (120), and administration of Fexofenadine significantly reduces serum IL-31 levels in AD patients (47).

Nemolizumab, developed based on these findings, is a humanized monoclonal antibody against the IL-31 receptor A (IL-31RA), administered subcutaneously, involved in pruritus and inflammation in AD. In a 16-week double-blind Phase III trial, Japanese AD patients with moderate to severe pruritus, insufficiently controlled by topical agents, were treated with subcutaneous nemolizumab in addition to topical agents. The results showed a reduction in pruritus compared to placebo plus topical agents (NCT03985943) (121). Further investigation into the long-term efficacy and safety of nemolizumab revealed continuous improvement in pruritus, AD signs, and QoL for up to 68 weeks when combined with topical agents, with a favorable safety profile (122). In another clinical trial with AD patients, nemolizumab rapidly and sustainably improved skin signs of inflammation and pruritus, with maximum effects observed at 30 mg, and the safety profile of nemolizumab was within an acceptable range. Nemolizumab significantly and rapidly improved inflammation, pruritus, and sleep in patients with a baseline EASI ≥16 (NCT03100344) (123, 124). Moreover, oral JAK inhibitors, which are also under development, have shown very promising effects on chronic pruritus through the Janus kinase 1/2 signaling pathway, a pathway involved with IL-4, IL-13, and IL-31 (125).

In summary, IL-31 plays a crucial role in the pathophysiology of human AD, particularly in its role in pruritus, highlighting its importance in the condition.

## 4 Chronic lesion

In the chronic phase, not only Th2 cells but also Th22 and Th17 cells increase in number, leading to suppression of filaggrin expression by IL-4, IL-13, and IL-22, which further deteriorates the epidermal barrier function. Additionally, IL-4 and IL-13 suppress the production of AMPs, weakening the barrier function against microbes. Th17 and Th22 cells also produce IL-22, which induces epidermal thickening.

### 4.1 IL-22

IL-22 is a cytokine produced by adaptive Th17 and Th22 cells, natural lymphocytes including γδT cells and type 3 innate lymphoid cells (ILC3), as well as myeloid cells including neutrophils. It belongs to the IL-10 family of cytokines (126). IL-22 is known to induce keratinocyte proliferation, and its serum levels are elevated in AD, with Th22 cells infiltrating the skin lesions of AD (127, 128). Additionally, the IL-22 receptor (IL-22R) is expressed on epithelial cells, including keratinocytes, but not on immune cells (129), suggesting that IL-22 signaling plays a crucial role in barrier function (130).

#### 4.1.1 Roles in mouse AD model

In mouse AD models, *in vivo* injection of IL-22 into the skin induces keratinocyte proliferation and epidermal thickening (131). When antigen is applied to the skin of mice subjected to tape stripping, an alternative to scratching, an IL-22 response that promotes epidermal hyperplasia and keratinocyte proliferation is induced (132).

#### 4.1.2 Roles in human AD

In human AD, there is a significant increase in IL22 mRNA expression and IL-22-producing T cells in the skin lesions (133–135). Serum IL-22 levels are also elevated in AD patients (127, 128). *In vitro* application of IL-22 to keratinocytes results in proliferation, and reconstituted human epidermis in a 3D matrix thickens (136, 137). In a clinical trial involving *in vivo* administration of fezakinumab to patients with moderate to severe AD, the IL-22 high-expression group showed much stronger improvement in transcriptomics mean values compared to the IL-22 high-expression placebo group and the IL-22 low-expression group (138).

From the above, it is suggested that IL-22 is involved in the pathophysiology of AD. In clinical trials, fezakinumab has demonstrated efficacy in adult patients with moderate to severe AD, with good tolerability (NCT01941537) (139). Specifically, larger and more significant differences were observed in the severe AD patient group.

In conclusion, IL-22 plays a crucial role in the pathophysiology of human AD, particularly in moderate to severe AD, demonstrating its significance in this condition.

### 4.2 IL-17A/25

IL-17A is pivotal for skin immunity, especially in inflammatory skin conditions like psoriasis, and it plays a vital role in the body’s defense against microbial pathogens. This cytokine is produced predominantly by Th17 cells, a unique lineage of proinflammatory T helper cells crucial for both autoimmune diseases and the regulation of innate immunity in epithelial cells, including keratinocytes, which form most skin cells. IL-17A, along with other cytokines secreted by Th17 cells, boosts the production of AMPs by human keratinocytes, strengthening the skin’s defense mechanisms against microbial invaders (140).

IL-25, also known as IL-17E, is a member of the IL-17 cytokine family. It is primarily produced by epithelial cells like keratinocytes but is also known to be produced by other immune cells such as T cells, DC, and ILC2 (141). IL-25 binds to a heterodimeric receptor composed of IL-25R and IL-17 receptor B (IL-17RB), also known as IL-17RA. The cellular targets of IL-25 include a variety of cells, such as T cells, ILC2, myeloid cell populations, invariant natural killer T (NKT) cells, fibroblasts, epithelial cells, endothelial cells, and mesenchymal cells (141).

#### 4.2.1 Roles in mouse AD model

In murine models of AD, IL-17A has been found to mediate Th2-type immune responses, positioning IL-17A signaling as a potential therapeutic target in AD (142). Studies using mice have demonstrated that both IL-25 and IL-33 are crucial for the development of allergic dermatitis through the regulation of ILC2 (143). However, while skin-associated ILC2 responses and AD-like dermatitis in a murine AD model are critically dependent on TSLP signaling, they are not dependent on IL-25 signaling (35). This suggests that IL-25 derived from Th2 T cells could amplify allergic-type inflammatory responses by acting on other cell types (144). In murine models, the depletion of a specific type of neonatal-derived γδT cell from birth resulted in a spontaneous and pervasive form of AD that displayed many key features of human AD (145). In the Flaky tail murine model of AD-like dermatitis, IL-17A has been proven to be involved in the activation of macrophages that are in the process of adopting heterogeneous profiles of both M1 and M2 states in the skin (146). Lastly, IL-17A mediates Th2-type immune responses in murine models of AD (142).

This comprehensive analysis underscores the multifaceted role of IL-17A and IL-25 in skin immunity and AD, highlighting their potential as targets for therapeutic intervention.

#### 4.2.2 Roles in human AD

In the context of AD, studies have demonstrated a marked increase in IL-17A levels in the serum of both adults and infants diagnosed with the condition. Importantly, this elevation in IL-17A levels has been found to correlate positively with the severity of the disease (147), suggesting a potential role in disease progression. Furthermore, a study has revealed that the interplay between specific genetic factors, such as the coexistence of the GG genotype of IL-17A rs2275913 and a mutation in the filaggrin gene (2282del4), can significantly heighten the risk of AD, highlighting the complexity of IL-17A’s role in AD and underscoring the need for further research to fully elucidate its mechanisms of action and potential as a therapeutic target (148).

Within the skin affected by AD, there has been observed an upregulation of both IL-25 and its receptor IL-17RB (149). This finding implies a potential involvement of the IL-25 signaling pathway in the pathogenesis of AD, though the precise impact of IL-25 on the skin barrier remains largely undefined. *In vitro* experiments have produced mixed results; for instance, IL-25 was found to decrease the expression of filaggrin mRNA in human keratinocytes cultured under high calcium conditions (149, 150), but this effect was not observed under other conditions (151). Furthermore, activated human eosinophils and basophils have been shown to produce IL-25 *in vitro* (149, 152). IL-25 has also been implicated in the pathophysiology of pruritus, a hallmark symptom of AD, through its ability to increase the expression of the pruritogenic substance endothelin-1 in cultured keratinocytes from both mice and humans, via the ERK1/2 or JNK pathways (153). Additionally, the administration of a specific probiotic strain, Lactobacillus plantarum IS-10506, resulted in the suppression of IL-4 and IL-17, accompanied by an alleviation of AD symptoms (154), further supporting the potential involvement of IL-17 in the pathology of AD.

However, it is crucial to acknowledge that the role of IL-17A in AD is intricate and multifaceted. Clinical trials with therapeutics targeting IL-17 pathways, such as MOR106 (Anti-IL-17C) and Secukinumab, have not demonstrated sufficient efficacy in AD patients (NCT02594098) (155, 156). Based on the preceding discussion, it can be inferred that the therapeutic targeting of IL-17 in human AD appears to offer constrained possibilities.

### 4.3 IL-23p19, IL-12/23p40

IL-23 is a cytokine belonging to the IL-12 family, uniquely composed of a specific p19 subunit and a shared p40 subunit with IL-12 (157). This cytokine is produced by various cells, including epidermal LCs, DCs, macrophages, and keratinocytes (158–160). IL-23 receptor (IL-23R) expression is found on several immune cells such as LC, DC, NK cells, NKT cells, γδT cells, and Th17 cells (161–163). IL-23 plays a critical role in promoting the polarization of Th17 cells (164, 165) and is essential for inducing the expression of IL-22 (131, 166).

#### 4.3.1 Roles in mouse AD model

In the context of mouse AD models, IL-23 released from keratinocytes in response to endogenous TLR4 ligands upregulates endogenous IL-23 production in skin DC, which selectively express IL-23R. This, in turn, drives the IL-22 response in naïve CD4+ T cells, leading to epidermal thickening (132).

#### 4.3.2 Roles in human AD

In human AD, an upregulation of the Th17/IL-23 axis has been demonstrated (142, 167). IL-23 is released in human skin after scratching and polarizes human skin DC to drive the IL-22 response (132). Risankizumab, an antibody that binds to the p19 subunit of IL-23, inhibiting its action (168), has been approved for the treatment of moderate to severe plaque psoriasis, active psoriatic arthritis, and moderate to severe active Crohn’s disease in adults.

Despite these findings, IL-23, and by extension the IL-17/23 axis, appears to be an insufficient therapeutic target for AD. This is supported by clinical trials conducted with risankizumab (an anti-IL-23A Ab) in AD patients aged 12 and older (NCT03706040) (169), as well as with ustekinumab (an anti-IL-12/23p40 Ab) in adult AD patients (NCT01945086), both of which did not demonstrate efficacy. These results suggest that while IL-23 is implicated in the pathophysiology of inflammatory diseases, including AD, targeting the IL-17/23 axis may not be an adequate strategy for AD treatment.

## 5 Discussion and conclusion

**FIGURE 2:**
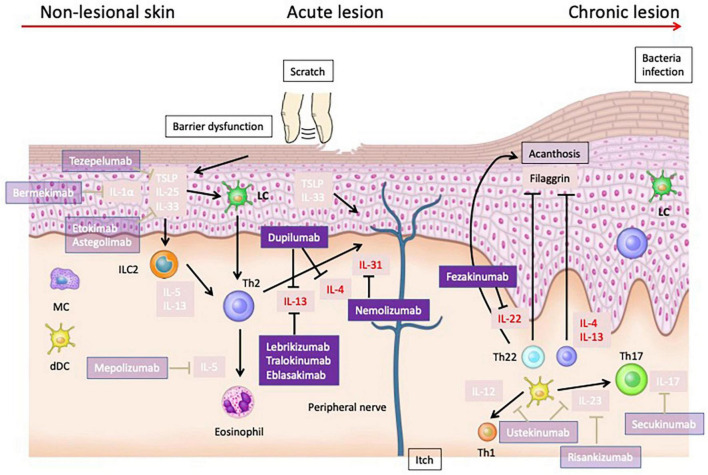
Cytokines that have been validated as therapeutic targets in human atopic dermatitis. Dupilumab, which targets IL-4 and IL-13, has shown efficacy in treating moderate to severe AD. Nemolizumab, targeting IL-31RA, effectively reduces pruritus in AD patients. Fezakinumab, targeting IL-22, have demonstrated promising results, particularly in severe AD cases. IL-33 has been identified as a potent activator of immune cells, exacerbating AD in murine models and correlating with disease severity in human patients. However, treatments targeting IL-33 have not shown sufficient efficacy in clinical trials. Similarly, TSLP, integral to type 2 immune responses, induces dermatitis in animal models and is elevated in human AD, yet clinical treatments like tezepelumab exhibit limited efficacy. Therapies targeting IL-1α, IL-5, and IL-17 also failed to achieve sufficient efficacy in clinical trials.

The pathogenesis of AD is complex and multifactorial, involving immune response dysregulation, compromised barrier function, and pruritus. Cytokines are pivotal in this process, with Th2, Th22, and Th17 cells contributing to the disease’s progression. IL-4 and IL-13, both Th2 cytokines, are key players in atopic inflammation, exacerbating epidermal barrier dysfunction, pruritus, and promoting type 2 immune deviation (170). IL-31, a pruritogenic cytokine, is produced by type 2 T cells and amplifies the IL-31-mediated sensory nerve signal (170). IL-22, produced by Th22 cells, mediates keratinocyte proliferation, epidermal hyperplasia, and antimicrobial protein production, and is implicated in the pathogenesis of atopic dermatitis (171). The efficacy of targeted treatments such as Dupilumab, Nemolizumab, and Fezakinumab in clinical trials underscores the importance of IL-4, IL-13, IL-31, and IL-22 as therapeutic targets in both acute and severe phases of AD. However, challenges remain, as treatments targeting IL-33, TSLP, IL-1α, IL-5, and IL-17 have shown limited success in clinical trials (Figure 2). This disparity between therapeutic effectiveness in murine models and human patients highlights the need for a nuanced understanding of AD’s pathophysiology. JAK inhibitors have shown promising results in the treatment of AD, with improvements in objective and subjective scoring indices observed in patients receiving both topical and oral formulations (172, 173). They have been associated with higher rates of achieving EASI75, Investigator’s Global Assessment response, and pruritus numerical rating scale response (173). However, they also carry a higher risk of treatment-emergent adverse events (173). Upadacitinib and abrocitinib, both selective JAK1 inhibitors, have been identified as effective and well-tolerated agents for moderate-to-severe atopic dermatitis (174). Despite these positive findings, further research is needed to establish the long-term efficacy and safety of JAK inhibitors in atopic dermatitis (175). The strength of this paper lies in its comprehensive examination of the roles of various cytokines in AD, taking into account unpublished negative trial data from the ClinicalTrials.gov database. A limitation, however, is the inability to conclusively determine whether the ineffectiveness of certain cytokine targets in trials is due to the drug itself or the unsuitability of the cytokine as a therapeutic target for AD.

Future treatment strategies must consider the differences between mouse model analyses and clinical trials differences to effectively address the diverse manifestations of AD.

## Author contributions

YY: Writing – original draft. CN: Writing – review and editing. AO: Writing – review and editing.
